# Genome Editing for Sustainable Agriculture in Africa

**DOI:** 10.3389/fgeed.2022.876697

**Published:** 2022-05-12

**Authors:** Leena Tripathi, Kanwarpal S. Dhugga, Valentine O. Ntui, Steven Runo, Easter D. Syombua, Samwel Muiruri, Zhengyu Wen, Jaindra N. Tripathi

**Affiliations:** ^1^ International Institute of Tropical Agriculture (IITA), Nairobi, Kenya; ^2^ International Maize and Wheat Improvement Center (CIMMYT), Texcoco, Mexico; ^3^ Kenyatta University, Nairobi, Kenya

**Keywords:** agriculture, genome editing, CRISPR/Cas, African crops, regulatory policies

## Abstract

Sustainable intensification of agriculture in Africa is essential for accomplishing food and nutritional security and addressing the rising concerns of climate change. There is an urgent need to close the yield gap in staple crops and enhance food production to feed the growing population. In order to meet the increasing demand for food, more efficient approaches to produce food are needed. All the tools available in the toolbox, including modern biotechnology and traditional, need to be applied for crop improvement. The full potential of new breeding tools such as genome editing needs to be exploited in addition to conventional technologies. Clustered regularly interspaced short palindromic repeats/CRISPR-associated protein (CRISPR/Cas)-based genome editing has rapidly become the most prevalent genetic engineering approach for developing improved crop varieties because of its simplicity, efficiency, specificity, and easy to use. Genome editing improves crop variety by modifying its endogenous genome free of any foreign gene. Hence, genome-edited crops with no foreign gene integration are not regulated as genetically modified organisms (GMOs) in several countries. Researchers are using CRISPR/Cas-based genome editing for improving African staple crops for biotic and abiotic stress resistance and improved nutritional quality. Many products, such as disease-resistant banana, maize resistant to lethal necrosis, and sorghum resistant to the parasitic plant Striga and enhanced quality, are under development for African farmers. There is a need for creating an enabling environment in Africa with science-based regulatory guidelines for the release and adoption of the products developed using CRISPR/Cas9-mediated genome editing. Some progress has been made in this regard. Nigeria and Kenya have recently published the national biosafety guidelines for the regulation of gene editing. This article summarizes recent advances in developments of tools, potential applications of genome editing for improving staple crops, and regulatory policies in Africa.

## Introduction

The greatest challenge in agriculture is to feed the growing population and mitigate the negative impact of climate change. Agriculture production needs to be doubled to feed the increasing global population projected to grow from 7.5 billion to 9.8 billion in 2050 (UNDESA, 2017). Africa’s population will double by 2050, making food security the main challenge for Africa (UNDESA, 2017). The biggest global challenges are producing more food with the same or less land and water, improving nutrition, and helping farmers adapt to climate change (Searchinger et al., 2019). The world can only meet its future food needs by harnessing scientific agriculture innovation.

The adverse impacts of climate change are already being felt in the form of increasing temperatures, weather variability, and invasive crops and pests. Some reports provide evidence to show the effects of climate change on agriculture production (Iizumi et al., 2018). The global yields of several grain crops such as maize, wheat, and soybeans have decreased due to climate change. The extreme climate is anticipated to have harmful influences on plant agronomic traits, pathogens and pests, and soil fertility, affecting crop productivity (Dhanker and Foyer, 2018). In Africa, climate change is predicted to negatively impact the food system due to extensive dependence on rainfed agriculture and the dominance of subsistence farming (Manners et al., 2021). Smallholder farmers in Africa are mainly dependent on root, tuber, and banana crops. A significant impact of variation in temperature and rainfall is reported on banana yields, mainly in the regions where the crop is cultivated with no or minimal irrigation (Sabiiti et al., 2016). Strategies need to be developed for African crops to adapt to extreme changes in climate, particularly drought.

Sustainable agriculture is vital for accomplishing food and nutritional security, as we are aware of the United Nations’ Sustainable Development Goals (SDGs) 2030 and addressing the rising concerns of climate change. Broad-ranging support is required for agricultural improvements in Africa, including recognizing farmer needs, high-yielding varieties, widely available and affordable planting material, and access to markets. Sustainable intensification of farming systems in developing countries, particularly in Africa, is essential to meet this growing global food demand (Tilman et al., 2011). Sustainable intensification of crop productivity requires agriculture innovation. There is an urgency to close the yield gap in staple crops and enhance food production to feed the world (Pixley et al., 2019).

**FIGURE 1:**
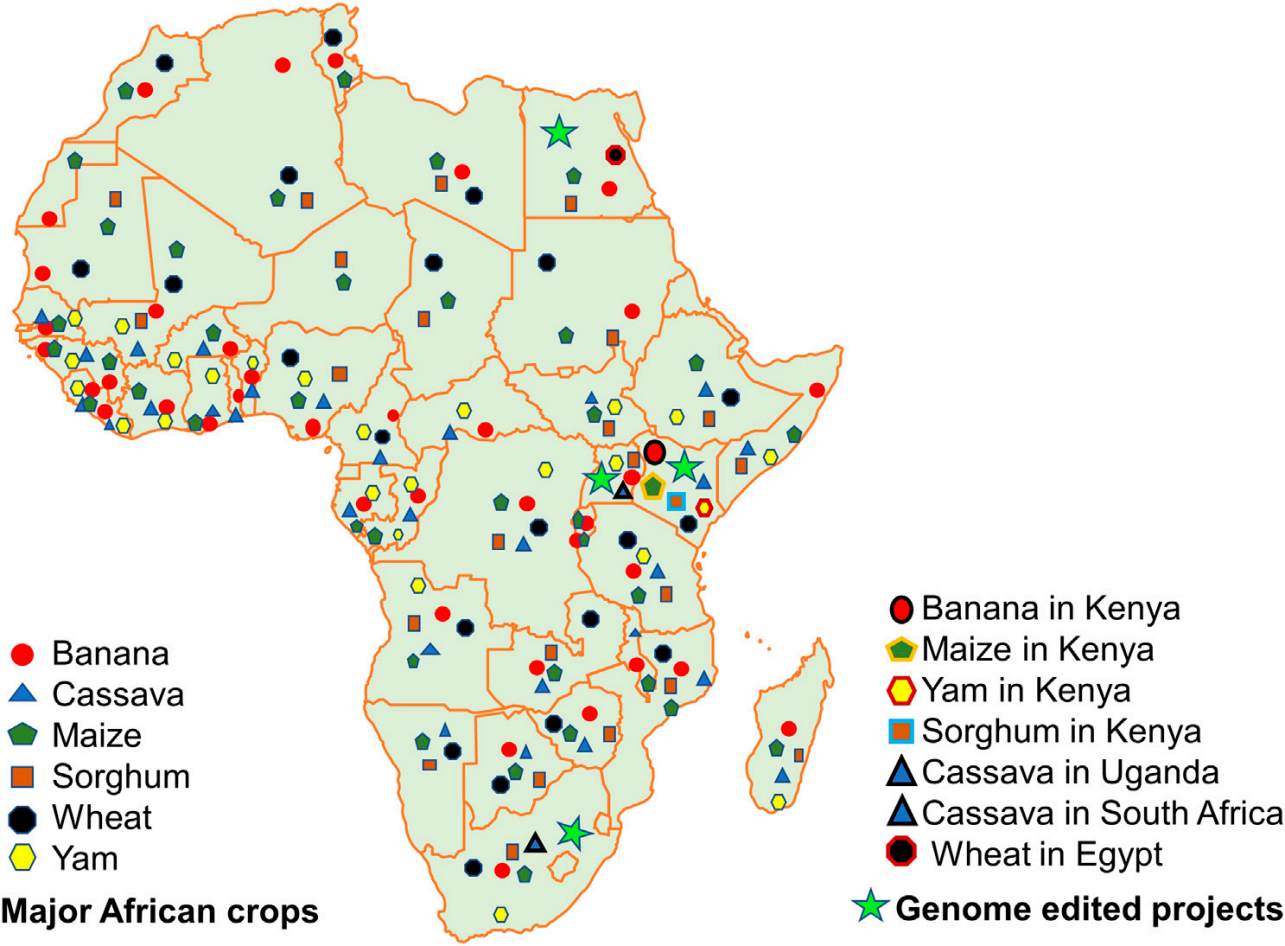
Map of Africa showing the major staple food crops addressed in this article. Also indicated are the countries where genome-edited projects are being implemented in Africa (based on the information from Karembu, 2021).

In order to meet the increasing demand for food, more efficient approaches to produce food are required. The full potential of new breeding tools such as genome editing needs to be exploited in addition to conventional technologies (Pixley et al., 2019). Genome editing technologies are simple, precise, and accurate, allowing targeted manipulation of the plant genomes, thereby speeding up the breeding efforts for developing improved crop varieties. Genome editing has the potential to reduce inputs such as fertilizers, pesticides, *etc*., increase yields, improve nutrition, and develop climate-resilient crops. Intensive efforts are underway; however, little has gone up to commercialization. Since the discovery of CRISPR/Cas9 technology, several Africa experts have been using this technology for crop improvement (Karembu, 2021). This article summarizes recent advances and highlights the progress in the CRISPR/Cas9-based genome editing efforts with major staple food crops grown in several countries in Africa (Figure 1).

## General Overview of Genome Editing Tools

Genome-editing technologies using site-directed nucleases (SDNs) have become powerful tools for modifying plant genomes. These tools, including meganucleases, zinc-finger nucleases (ZFNs), transcription activator-like effector nucleases (TALENs), and clustered regularly interspaced short palindromic repeats/CRISPR-associated protein (CRISPR/Cas), achieve precise genetic modifications by inducing targeted DNA double-strand breaks (DSBs). The DSB may then be repaired by either non-homologous end-joining (NHEJ) or homology-directed repair (HDR), depending on the cell cycle stage and the presence or absence of a repair template containing homologous terminal regions (Tripathi et al., 2020; Ntui et al., 2021). Mechanisms involving HDR have been achieved in crops discussed here, including cassava and maize (Hummel et al., 2018; Gao et al., 2020b; Veley et al., 2021). Additionally, base and prime editing have been achieved in some of the cereal crops, such as maize (Zong et al., 2017).

Meganucleases, encoded by mobile genetic elements or introns, are naturally occurring, compact DNA cleavage enzymes that recognize long (∼20 base pairs) DNA targets. ZFNs are based on a custom-designed Cys_2_-His_2_ zinc-finger protein and the FokI restriction endonuclease cleavage domain. TALENs are derived from TALEs of bacteria and consist of an amino-terminal TALE DNA-binding domain fused to a carboxy-terminal FokI cleavage domain. CRISPR/Cas9 is derived from the adaptive immune system of *Streptococcus pyogenes*. Among all the genome editing tools, CRISPR/Cas9 has become the most popular approach due to its simplicity, efficiency, specificity, easy to adapt, and capability of multiplexing traits.

The main components of CRISPR/Cas9 are the guide RNA (gRNA) and the Cas9 nuclease. The Cas9/gRNA complex recognizes target DNA and the protospacer adjacent motif (PAM) sequence and begins editing the DNA upstream of the PAM segment. The PAM is a three-nucleotide sequence that serves as a recognition site for Cas9 to start editing upstream. It is usually NGG or NAG, where N is any nucleotide. The gRNA, consisting of a scaffold and a user-defined spacer sequence of about 20 nucleotides, directs the Cas9 to create precise double-stranded breaks (DSBs).

Other Cas proteins such Cas12a (Cpf1) and Cas13a have also been used in several crops. Cas12a is a class 2, type V-CRISPR system, which harbours a RuvC domain, possesses crRNA biogenesis RNase and single-strand DNase activities and recognizes a T-rich PAM, TTN/TTTN/TTTV (N = A/T/C/G; V = A/C/G) (Bandyopadhyay et al., 2020). Cas13a is a ribonuclease of class 2 type VI-A that targets and cleaves single-stranded RNA (ssRNA) molecules in the phage genome (Aman et al., 2018). It does not require a PAM and can be used to detect RNA viruses.

Based on the repair mechanism, the editing can lead to three different outcomes: site-directed nuclease-1 (SDN1), where after a cut by the CRISPR/Cas9 of the host DNA, non-homologous end joining (NHEJ) introduces indel mutations during repair leading to gene knockout, gene silencing, and gene inactivation; SDN2, which involves template-mediated sequence alteration to change the gene function; and SDN3, where a DNA fragment is inserted or replaced at a precise location in the genome (Podevin et al., 2013; Svitashev et al., 2015). SDN1 is like mutations obtained through chemical mutagenesis, irradiation, or spontaneous natural mutations and are not regulated as genetically modified organisms (GMO) in many countries (Tripathi et al., 2020).

Base and prime editing techniques have also recently been developed. Base editing requires the fusion of a DNA deaminase to dCas9 (dead Cas9) to generate a base editor that enables a single base substitution without involving a DSB. In this system, a guide RNA binds to the target DNA, and during the process of the base pairing of the guide RNA with the target DNA, a DNA bubble having a short segment of ssDNA is created. The deaminase enzyme then modifies the targeted base of the ssDNA, and depending on the DNA deaminases, C:G-to-T:A or A:T-to-G:C substitution could be achieved. Prime editing uses the exact mechanism as classical CRISPR/Cas9 systems, mediating DNA base pair substitutions, small insertions, or small deletions (indels), but does not induce a DSB and does not require a donor template (Matsoukas, 2020; Chen et al., 2021). It requires a longer-than-usual gRNA, known as pegRNA, and a fusion protein consisting of Cas9 H840A nickase fused to an engineered reverse transcriptase enzyme to edit the genome. Since base editing and prime editing do not require a DNA donor template, they might be considered as SDN1 type of gene editing, which can be treated like non-GMO products and do not require biosafety regulations similar to transgenic products.

To expand CRISPR/Cas9 functions, a CRISPR tool known as CRISPR activation (CRISPRa) was developed. CRISPRa uses a modified version of Cas9 without the endonuclease activity (dead Cas protein; dCas) with added transcriptional activators or repressors *VP64*, *VPR*, or *Mxi1* to enhance or repress the expression of the desired gene (Chen and Qi, 2017). Fusion of dCas9 with activation or repression domains allows specific and efficient transcriptional regulation of any gene without introducing any mutations in the endogenous gene.

## Potential Applications for Improvement of Staple Crops in Africa

### Banana

Banana, including plantain, is an important staple food crop and a source of income for millions of resource-poor farmers in Africa. The crop is primarily grown by smallholder farmers for domestic consumption and local or regional markets, and less than 15% enter the international markets. Africa produces one-third of the global bananas, with East Africa the leading producer accounting for about 40% of the total African production (FAOSTAT, 2020). East Africa is the largest banana-growing and -consuming region, with the most substantial consumption at 220–460 kg per person annually, which is six times Africa’s average and 15 times the world’s average (Ainembabazi et al., 2015). According to Food and Agriculture Organization (FAO) data, over 60% of the total banana cultivation across Africa is from East Africa (FAOSTAT, 2020). Still, there is a huge yield gap for banana and plantain production in Africa. Banana and plantain yield in most African countries is among the lowest globally. The average yield for banana and plantain is 12 tons/ha and 5.5 tons/ha annually (FAOSTAT, 2020), which is relatively low compared to the potential yield of 70 tons/ha/year for banana and 35 tons/ha/ton for plantain. This yield gap is because the crop is vulnerable to several diseases, mainly when many diseases are present together in the same region. The most significant diseases are bacterial [banana Xanthomonas wilt (BXW) caused by *Xanthomonas campestris* pv. *musacearum*, moko and bugtok disease, caused by *Ralstonia solanacearum,* and blood disease, caused by *Ralstonia syzygii* subsp. *Celebesensis*]*,* fungal (black Sigatoka caused by *Pseudocercospora fijiensis*, Fusarium wilt, commonly known as Panama disease, caused by *Fusarium oxysporum* f. sp. *cubense*), and viral (banana bunchy top disease, and banana streak disease) (Blomme et al., 2017; Tripathi et al., 2020).

The CRISPR/Cas9-based genome editing tool has successfully been established in banana using the *phytoene desaturase* (*PDS*) gene (Kaur et al., 2018; Naim et al., 2018; Ntui et al., 2020). PDS is a key enzyme in the carotenoid biosynthesis pathway and is widely used as a visual marker to optimize genome editing protocols. It encodes a crucial enzyme that converts phytoene to ζ-carotene in the pathway. Functional disruption of *PDS* produces albino and dwarf plantlets. Kaur et al. (2018) established genome editing in a banana for the cultivar “Rasthali” (AAB genome). The authors used a single gRNA to generate mutations in the *PDS* gene, producing albino phenotypes, but with only 59% mutation efficiency. Further, Naim et al. (2018) reported mutation of the *PDS* gene in the cultivar “Cavendish Williams” (AAA genome) with a 100% editing efficiency using polycistronic gRNAs. Later, Ntui et al. (2020) reported 100% mutation efficiency in banana cultivar “Sukali Ndiizi” (AAB genome) and plantain cultivar “Gonja Manjaya” (AAB genome) by multiplexing gRNAs targeting the *PDS* gene. As an alternative to *PDS* gene, Zorrilla-Fontanesi et al. (2020) edited *RP43/CHAOS39* as a visual marker to optimize genome-editing procedures in banana. *RP43/CHAOS39* is a gene that encodes the chloroplast signal recognition particle (cpSRP) machinery. The *CHAOS39*-edited banana plants had pale-green phenotypes and normal growth. The CRISPR/Cas9-based genome editing techniques established in various laboratories are currently being used to produce edited banana with important agronomic traits such as disease resistance and nutrient enhancement (Kaur et al., 2020; Tripathi et al., 2021; Tripathi et al., 2019b; Tripathi et al., 2020).

**FIGURE 2:**
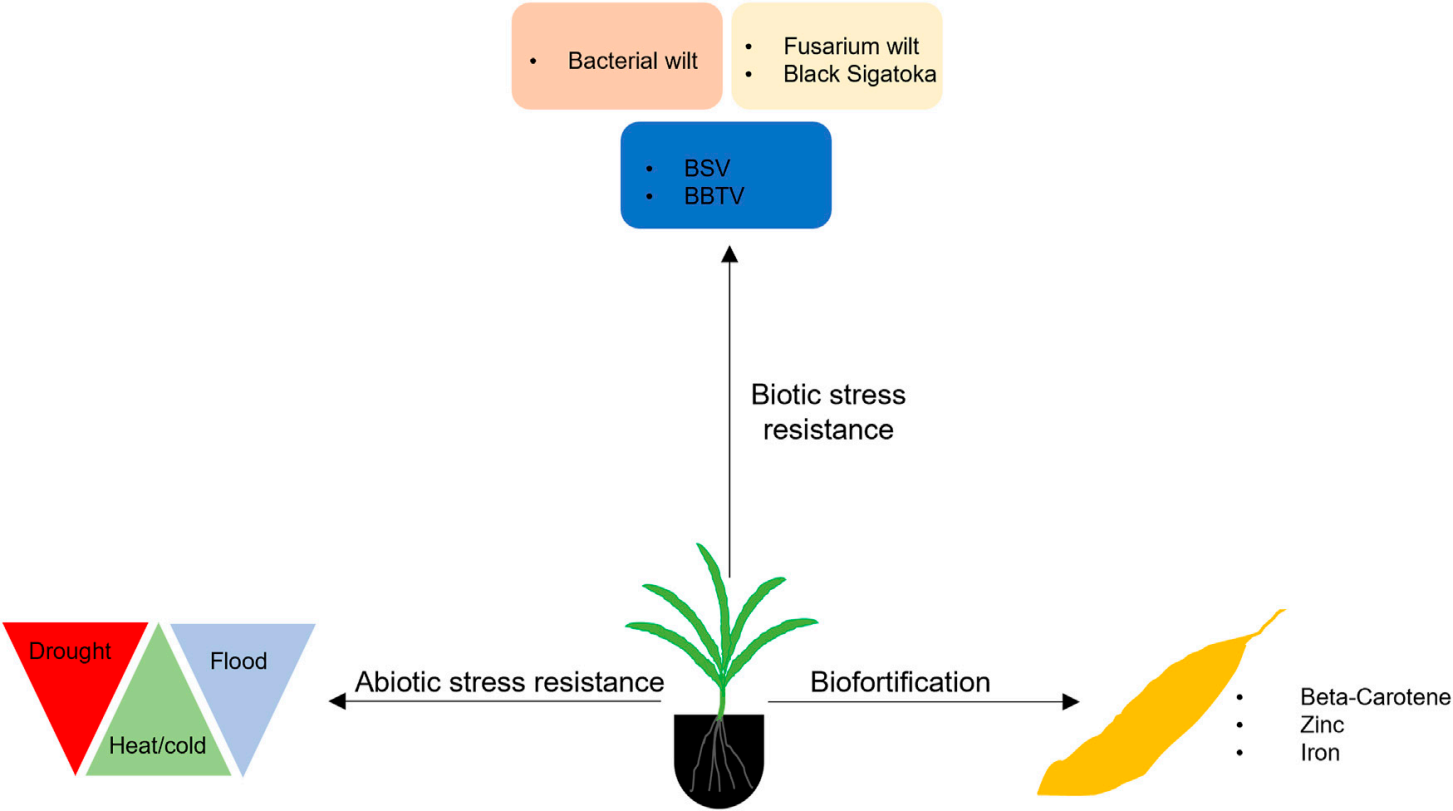
Application of genome editing in banana for developing improved varieties with biotic and abiotic resistance and enhanced nutrition. BSV, Banana Streak Virus; BBTV, Banana Bunchy Top Virus.

The CRISPR/Cas9-based genome editing established for banana is paving the way for functional genomics allowing the identification of genes associated with biotic and abiotic stress tolerant traits and nutrition enhancement, which could be used to improve banana for enhanced nutrition and adaptation to a changing climate (Figure 2).

#### Disease Resistance

Targeted genome editing technology such as CRISPR/Cas9 can be used efficiently to develop disease-resistant bananas. The accessibility of full banana genome-sequences and the CRISPR/Cas9 gene-editing systems has made it easy to generate disease-resistant banana by precisely knocking out the endogenous genes (Ntui et al., 2020). Several susceptibility genes associated with bacterial resistance have been identified and targeted for editing in several plants (Tripathi et al., 2020).

Researchers at the International Institute of Tropical Agriculture (IITA) are developing banana resistant to BXW disease. All the cultivated varieties are susceptible to BXW disease. There is no known source of resistance against the bacterial pathogen within *Musa* germplasm except for the wild-type diploid banana progenitor “*Musa balbisiana*” (Tripathi et al., 2019a). The knowledge of resistance mechanisms in wild-type banana against a bacterial pathogen can be utilized for developing resistant varieties through the editing of genes related to susceptibility and/or negative regulation of plant immunity or activating the defense genes. To identify the *Musa* genes for developing the BXW-resistant varieties, we investigated the molecular basis of disease resistance in banana progenitor *Musa balbisiana*. The comparative transcriptomic analysis of *Musa balbisiana*, a wild-type progenitor resistant to BXW, and the highly susceptible banana cultivar “Pisang Awak” inoculated with *Xanthomonas campestris* pv. *musacearum*, identified a couple of susceptibility genes, nutrient transporters, E3 ubiquitin-protein ligase, pathogen-associated molecular patterns, receptor kinases, antimicrobial peptides, resistance proteins, and defense signaling genes associated with BXW resistance (Tripathi et al., 2019a).

Another approach we are using is to transfer the knowledge on bacterial pathogen resistance from other plant species to the banana. We have demonstrated the knockout of *Musa Downy mildew resistance 6* (*Musa DMR6*) gene in edited banana cultivar “Sukali Ndiizi” enhanced resistance to BXW disease (Tripathi et al., 2021). *DMR6* encodes a 2-oxoglutarate Fe (II)-dependent oxygenase (2OGO) and was characterized as a negative regulator of plant defense by hydrolyzing the plant defense signaling molecule salicylic acid (Zhang et al., 2017). It is upregulated during pathogen infection (Low et al., 2020). We performed a phylogenetic analysis of the 2-oxoglutarate Fe(II)-dependent oxygenase gene family with five plant species, including *Musa acuminata*, *Musa balbisiana*, *Arabidopsis thaliana*, *Solanum lycopersicum*, and *Nicotiana tabacum* (Tripathi et al., 2021). *AtDMR6* orthologues were identified in *Musa* spp., and one of the *MusaDMR6* orthologue (Ma04_p20880.1) was selected for analysis as a putative candidate. Banana mutants were generated targeting the *MusaDMR6* orthologue, and mutations were confirmed by sequencing. The *Musadmr6* mutants generated by a multiplexed CRISPR/Cas9 construct were evaluated in the greenhouse for resistance to BXW disease. The edited events showed enhanced resistance to BXW, and no morphological abnormalities were observed (Tripathi et al., 2021).

Banana streak virus (BSV), a dsDNA virus belonging to badnaviruses, integrates into the host plant genome creating a significant challenge in banana breeding and germplasm movement. A CRISPR/Cas9 system was used to inactivate the integrated endogenous BSV (eBSV) by targeting all three open reading frames (ORF) of the virus (Tripathi et al., 2019). The regenerated genome-edited plants of “Gonja Manjaya” showed targeted mutations in the integrated eBSV sequences in the host genome. As the eBSV gets activated into infectious viral particles under stress conditions leading to the development of disease symptoms, the genome-edited plants were water stressed in the greenhouse. Most of the mutants remained asymptomatic compared to the control non-edited plants under water stress conditions, confirming the silencing of the reactivation of eBSV into infectious viral episomal proteins.

Several banana researchers are developing banana varieties resistant to fusarium wilt disease. In the 1950s, Fusarium wilt race 1 outbreak wiped an entire “Gros Michel” farm and was replaced by “Cavendish” varieties, which currently cover about 90% of export markets (Ploetz, 2015). Management of diseases has mostly been achieved using chemical and resistant cultivars (Tripathi et al., 2019b). However, the evolution of new ecotypes makes using new breeding tools such as genome editing to generate resistance a continuous requirement. As of now, no work has been published on the use of genome editing in a banana for resistance to fungal pathogens. However, editing of susceptibility genes such as *Mildew resistance locus O* (*MLO*), *LATERAL ORGAN BOUNDARIES* (*CsLOB1*), *DMR6*, *ERF922*, amongst others, have been shown to confer resistance to fungal diseases (Jia et al., 2016; Wang et al., 2016; Peng et al., 2017; de Toledo Thomazella et al., 2021; Li et al., 2022). These approaches can be adopted in banana to generate resistance against fusarium wilt and black Sigatoka. Currently, researchers at IITA are testing the edited banana with S gene knockout in the greenhouse for resistance against fusarium wilt disease.

#### Nutrition Enhancement

Biofortification is a cost-effective approach to increase vitamins and minerals in food crops and ameliorate malnutrition (hidden hunger). Vitamin A deficiency, one of the most dominant micronutrient deficiencies, affects people in Africa. Genome editing has significant potential to enhance micronutrient bioavailability in crops through biofortification.

Some progress has been reported in enhancing nutrients such as iron, zinc, carotenoid, and amino acids in different crops like camelina, grape, potato, rapeseed, rice, sweet potato, tomato, and wheat using CRISPR/Cas9 technology targeting various genes (Liu et al., 2021). Recently, Kaur et al. (2020) applied CRISPR/Cas9 technology to increase β-carotene content in the Cavendish cultivar “Grand Naine” by editing the *lycopene epsilon-cyclase* (*LCY*ε) gene. They showed that the edited lines had enhanced accumulation of β-carotene content up to 6-fold (∼24 μg/g) in the fruit pulp compared to the unedited plants. The editing of genes regulating zinc, iron, amino acids, and other nutrients could also be targeted in banana to increase the nutrient contents.

#### Plant Architecture

Most of the cultivated banana varieties are very tall, and some of them are challenged by the weak lodging and severely damaged during storms. Dwarf and semi-dwarf varieties are better for crop growth and harvesting. Therefore, researchers are trying to develop semi-dwarf and dwarf banana varieties. Gibberellin (GA) is a critical gene determining plant height and mutations in its biosynthesis genes usually produce dwarf phenotypes. Shao et al. (2020) demonstrated that CRISPR/Cas9 tool could be applied to develop semi-dwarf plants by editing the *Musa acuminata gibberellin 20ox2 (MaGA20ox2*) gene, disrupting the GA pathway in banana cultivar “Gros Michel.”

#### Delay Ripening

Banana is a climacteric fruit with a soft texture upon ripening. Generally, banana ripens fast and start decaying within a week. The delay in the ripening of banana fruit can enhance its limits on storage, transportation, and marketing and reduce postharvest losses. CRISPR/Cas9-mediated genome editing of banana targeting the *aminocyclopropnae-1-carboxylase oxidase (MaACO1*) demonstrated delayed ripening, enhancing the shelf life of fruit (Hu et al., 2021). The edited banana fruits showed reduced ethylene synthesis and extended shelf life under natural ripening conditions.

### Cassava

Cassava is an important crop cultivated for its edible tuberous roots and minimally for its leaves in tropical and subtropical regions. Its production in Africa is mainly constrained due to two viral diseases [Cassava Mosaic Disease (CMD) and Cassava Brown Streak Disease (CBSD)] and a bacterial disease [Cassava Bacterial Blight (CBB)]. CMD, a major cassava disease, is caused by a group of related whitefly transmitted bipartite, single-stranded circular Geminivirus variants called Cassava Mosaic Viruses (CMV). The variants include East Africa and South African Cassava Mosaic and others named after the geographical regions they are endemic to (Legg, 2008). The CMD manifests itself in cassava through mosaic-like chlorosis, leaf twisting, and distortion, a combination of which results in reduced photo assimilation and root yield (Legg, 2008). The CMD disease is devastating and can result in yield losses of between 20 and 100%. Improvement of cassava against CMD has consistently been achieved through conventional breeding approaches that have been very successful, resulting in disease-resistant cultivars. There are three known mechanisms for CMD resistance in cassava; the CMD1 (recessive QTL), CMD2 (dominant gene CMD2), and CMD3 (recessive QTL) (Hahn et al., 1980). A recent report of CMD2 loss of resistance in cassava plants that have gone through tissue culture is worrying (Beyene et al., 2016; Chauhan et al., 2018).

The second major viral disease of cassava is CBSD, caused by Cassava Brown Streak Virus (CBSV) and its variants Uganda Cassava Brown Streak Virus (UCBSV) (Mohammed et al., 2016). The CBSV and UCBSV are positive single-stranded RNA viruses of the genus *Ipomovirus* family Potyviridae*.* Unlike CMD, which reduces yield, CBSD destroys roots leading to total harvest loss (Gomez et al., 2019). Approaches that can complement traditional breeding in quickly introducing resistance to CMD and CBSV would go a long way in ensuring improved resistance to these destructive viral diseases.

CBB is the major bacterial disease of cassava caused by *Xanthomonas axonopodis* pv. *manihotis* (Xam). The disease has a global distribution, with reports of its presence in all the major cassava growing areas (Lamptey et al., 1998; Oduro et al., 2004). Predictions of yield losses resulting from CBB are difficult owing to the high environmental influence on the disease’s severity (Banito et al., 2008). However, it has been observed that CBB can result in up to 100% loss in productivity during high humidity and moisture, conditions that allow maximum severity (López and Bernal 2012). Efforts to map the sources of CBB resistance in different cultivars have been carried out mainly through QTLs (López et al., 2007; López and Bernal, 2012; Soto Sedano et al., 2017). Due to high levels of environmental influence on CBB severity, no major QTLs have been mapped yet. Most of the QTLs account for less than 20% resistance. Genome editing approaches could complement conventional breeding efforts in enhancing resistance to CBB.

The revolutionary genome editing has opened opportunities for the improvement of cassava and could complement conventional breeding. CRISPR/Cas9-based genome editing in cassava was successfully established by targeting the *PDS* gene (Odipio et al., 2017). The Cas9 and two gRNAs targeting exon 13 of the cassava *PDS* (*MePDS*) were delivered into the friable embryogenic cells (FECs) of cassava (cv. 60444 and TME 204) *via Agrobacterium tumefaciens* (Odipio et al., 2017). The authors reported multi-allelic mutations in *MePDS* at a high frequency and set the stage for using this method in cassava. However, the main challenge in cassava remains the development of FECs, which is variety dependent; therefore, there is a need to improve this method. Approaches that may not require the generation of FECs through tissue culture would be an improvement. One such would be the *de novo* meristem induction system that does not require the long tissue culture process required in generating FECs (Maher et al., 2020). In a more recent study, another approach that transiently expresses CRISPR/Cas9 components in protoplasts has been reported in cassava (Chatukuta and Rey, 2020). However, this approach is restricted to the functional characterization of genes. The authors could not regenerate complete plantlets from the cassava protoplast. Therefore, there is a need to develop a cassava regeneration system using protoplast to develop improved varieties suitable for the farmers.

#### Disease Resistance

Development of resistance to the two major devastating viral diseases of cassava, CMD and CBSD, has been attempted using CRISPR/Cas9 technology (Gomez et al., 2019; Mehta et al., 2019). To generate plants with resistance to CMD, Mehta et al. (2019) targeted the viral AC2 gene coding for the multifunctional TrAP protein involved in gene activation, virus pathogenicity, and suppression of gene silencing, and the AC3 gene coding for the REn protein involved in replication enhancement using single gRNA. In this study, the transgenic cassava lines expressing *Cas9* gene along with a gRNA targeting the viral genome did not show resistance to CMD under greenhouse conditions. The authors further sequenced full-length viral amplicons from the infected transgenic and wild type plants. Different types of mutations in the viral genome were observed, including deletions within the gRNA target site that resulted in premature stop codon within the AC2 and AC3 ORFs. The indels were observed in viruses infecting all plants, including control wild-type plants. One major observation from this study was the introduction of histidine (H) to glutamine (Q) amino acid in the AC2 ORF of the viruses infecting the transgenic cassava plants. This H to Q mutation was a result of a single nucleotide (“T”) insertion that rendered the gRNA target impossible to bind and cleave. The authors observed about 33–48% of edited virus genomes evolve a conserved single nucleotide mutation in AC2 that confers resistance to CRISPR/Cas9 cleavage. The authors concluded that the CRISPR/Cas9 system may not be effective for developing resistance against Geminivirus as it may lead to virus evolution. However, this generalization has since been rebutted (Rybicki, 2019). The major argument in the rebuttal by Rybicki (2019) is that the mutation could have arisen spontaneously even in the wild-type virus rather than it being selected by CRISPR/Cas9 as detailed in Mehta et al. (2019). Rybicki (2019) further argues that resistance to other begomoviruses has been successfully demonstrated in other plants without resulting in Mehta’s observations (Baltes et al., 2015). The other major limitation of the Mehta et al. (2019) study is using only one gRNA that targeted one viral region (AC2). It would be ideal to experimentally target other viral ORFs and check if similar trends in virus evolution are observed.

The use of CRISPR/Cas9 to generate cassava resistant to the CBSD, the second major cassava virus, has also been attempted (Gomez et al., 2019). In this study, they used the fact that viruses in the potyviridae family use the eukaryotic translation initiation factor 4E (eIF4E) and novel cap-binding protein (nCBPs) to initiate cap-dependent messenger RNA translation. A CRISPR/Cas9 system targeting nCBPs was developed to introduce mutations that attenuated CBSD aerial symptoms in the mutants. This study only targeted the two nCPBs in cassava and multiplexing to target the other three eIF4Es could be done to achieve increased resistance.

The plant bacterial pathogens of the genus *Xanthomonas* promote pathogenicity by injecting effector proteins through the type III secretion system. Some of the injected proteins are transcriptional activator-like effectors (TALE), which control transcription in a manner similar to eukaryotic transcription factors (TFs) (Cox et al., 2017). The TALEs bind at specific promoter regions in the susceptibility genes called effector-binding elements (EBEs). These susceptibility genes are involved in sugar transport (SWEET genes), and their expression possibly provides energy source for the bacteria. Modification of *Xanthomonas* EBEs through genome editing has been observed to result in disease resistance (Xu et al., 2019; Li et al., 2020; Zafar et al., 2020). Similar efforts could be applied in cassava where promoters of susceptibility genes can be identified and modified to generate resistance lines. A recent report details efforts to develop CBB resistance through CRISPR/Cas9-based editing of the promoter of the cassava *MeSWEET10a* gene (Wang et al., 2022). The cassava mutants conferred enhanced resistance to CBB disease. The events also showed normal morphological and yield-related traits similar to the wild type.

#### Early and Synchronized Flowering

Conventional breeding in cassava is time-intensive, taking a minimum of 6 years to get to cultivar trials and even longer to release a cultivar. The breeding time is lengthened by variation in plant performance on the physiological status of the vegetative cutting and mostly by delayed and non-synchronized flowering of cassava breeding lines (Adeyemo et al., 2019). Approaches that can shorten the flowering time and synchronize flowering will go a long way in enhancing breeding and cultivar improvement in cassava (Ceballos et al., 2015).

Targeting flowering-related genes using CRISPR/Cas9 is a plausible approach that can be used to induce flowering in cassava. Silencing of flowering repressor using CRISPR/Cas9 has resulted in early flowering mutants in soybean (Han et al., 2019). In this study, CRISPR/Cas9 targeted a soybean flowering repressor gene *E1*, resulting in early flowering. The edited soybean line showed photoperiod insensitive flowering, allowing it to grow at different altitudes. Cassava, just like soybean, is a long-day crop and is dependent on extended photoperiod to flower. Mapping the major genes involved in photoperiod-dependent flowering in cassava could facilitate a study like Han et al. (2019) to develop photoperiod-insensitive cassava lines. Early flowering through CRISPR/Cas9 has also been achieved in Chinese cabbage (Jeong et al., 2019). This study used six gRNAs to target the *Flowering locus C (FLC)* in Chinese cabbage. The knockout obtained had early flowering as well as vernalization-independent phenotypes. Cassava has multiple *FLC* locus that needs to be experimentally characterized. The CRISPR/Cas9 editing approach by Jeong et al. (2019) is applicable in cassava for first functionally characterizing the *FLC* and target *FLC* versions involved in repressing flowering.

Induction of flowering through an inducible CRISPR/Cas9 system is a better option compared to constitutive overexpression. Inducible CRISPR/Cas9 systems are still under development, but recent progress has been made. One such CRISPR system takes advantage of a light-inducible heterodimerizing Cas9 protein (Polstein and Gersbach, 2015). Inducible systems would be ideal for overexpression of genes like FT in cassava which requires controlled expression. Overall, these studies point towards possible applications of CRISPR/Cas9 in the induction of flowering in cassava.

### Maize

Maize (*Zea mays* L.) is a staple food in sub-Saharan Africa, which, aside from providing nutrition and food security, supports the livelihoods of smallholder farmers (Nuss and Tanumihardjo, 2011; Boddupalli et al., 2020). Biotic and abiotic stresses underlie the gap between the potential and the harvested grain yield (Duvick, 2005). Maize grain yield in most African countries is among the lowest globally (Anon., 2018). Any further reduction by environmental stresses exacerbates food in security (Wulff and Dhugga, 2018).

Maize was one of the first crops where genome editing was used to introduce gene variants to improve corresponding traits, for example herbicide tolerance and grain biofortification (Shukla et al., 2009; Zhu et al., 1999; 2000). One of the first editing report involved using ZFNs to modify *INOSITOL PHOSPHOKINASE1* (*ZmIPK1*) by inserting *PAT* gene cassettes, resulting in herbicide tolerance and alteration of the inositol phosphate profile of developing maize seeds (Shukla et al., 2009). Later, meganucleases-based editing was developed and demonstrated using a derivative of I-CreI named LIG3::4, which recognizes a site upstream of the *LIGULELESS1* (*LG1*) gene solely in the genome of the maize inbred EXT (Gao et al., 2010). In 2015, Char et al. (2015) demonstrated the use of TALEN for editing maize for the glossy phenotype and reduced epicuticular wax in the leaves by knocking out the *GL2* gene. However, the extremely low frequency of the edited events posed a hurdle for this technology to go mainstream.

Following the development of CRISPR, genome editing in maize expanded rapidly. Svitashev et al. (2015) were the first to demonstrate CRISPR/Cas9 genome editing in maize by editing multiple genes such as *LG1*, the male fertility genes *MS26* and *MS45*, and the *acetolactate synthase* genes *ALS1* and *ALS2*. Waxy allele was deleted directly in elite maize lines using SDN1, which not only saved time and field resources, but the hybrids produced from those lines outyielded their counterparts produced by conventional introgression of the waxy allele (Gao et al., 2020a). Further, the production of DNA-free genome-edited maize by delivering Cas9/gRNA ribonucleoproteins (RNPs) into the plant cells was reported (Svitashev et al., 2016). Maize is one of the few crops where genes have been edited using all three scenarios: SDN1, SDN2, and SDN3 (Svitashev et al., 2015; Shi et al., 2017).

A potential challenge in genome editing of maize is the ability to transform elite lines, particularly those that are parents of commercial hybrids in Africa. This hurdle has been recently overcome by including genes for cell morphogenesis in the transformation plasmids (Lowe et al., 2016, 2018). Under a partnership, Corteva Agriscience and CIMMYT have used this technology to transform elite CIMMYT inbred lines from eastern Africa. The ability to transform tropical maize lines has paved the way for gene editing directly in the commercial lines.

**FIGURE 3:**
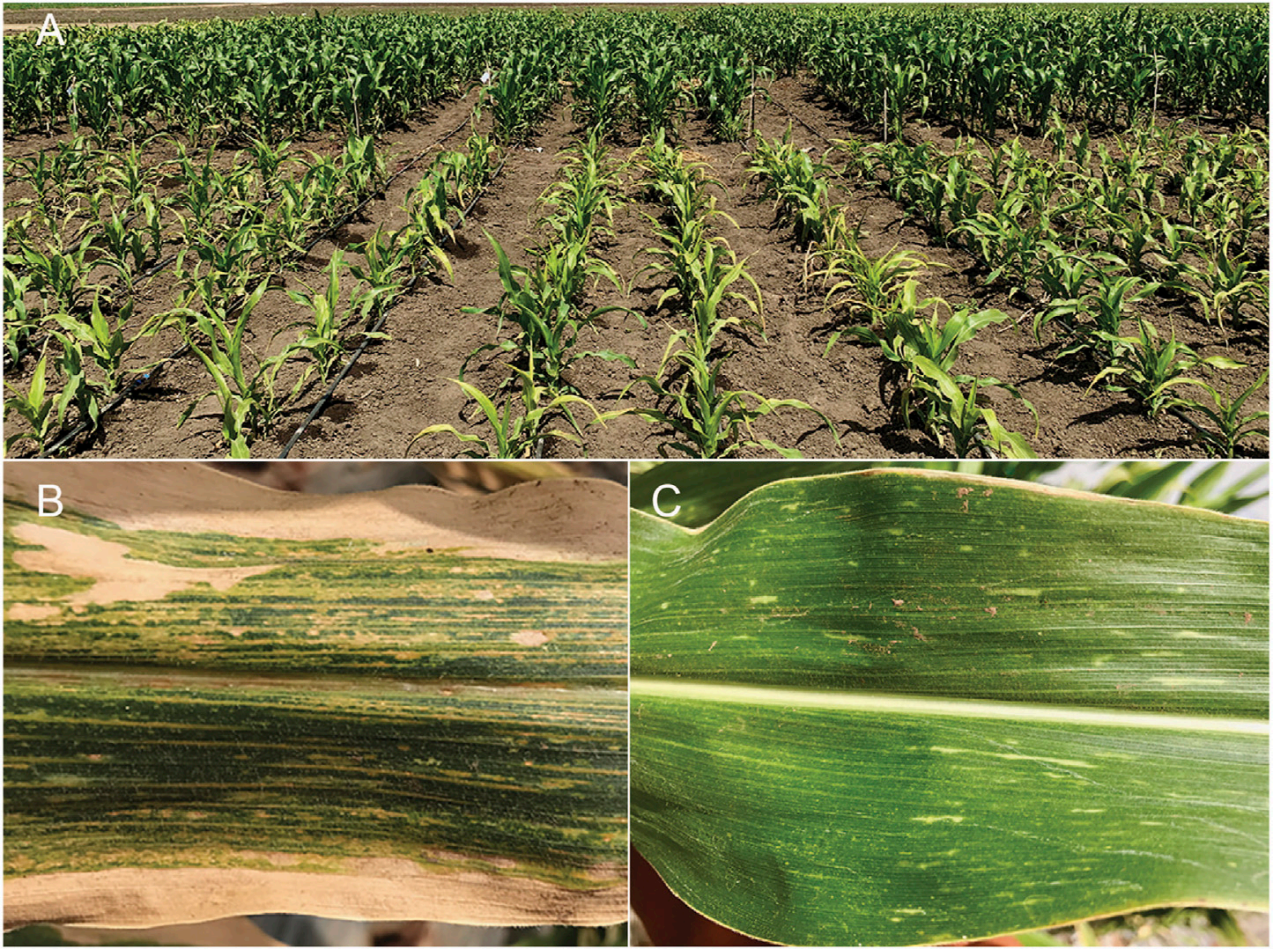
Plants resistant or susceptible to maize lethal necrosis (MLN) in Naivasha. Susceptible (in front) and resistant (in the back) plants 2 weeks after inoculation with a combination of MCMV and SCMV **(A)**. A closeup of the leaves from plants susceptible **(B)** or resistant **(C)** to MLN.

Maize lethal necrosis (MLN), a viral disease, has wreaked havoc in eastern Africa since it first appeared in Kenya in 2011 (Boddupalli et al., 2020). It was first reported in the USA some four decades ago and appeared in Kenya in 2011, from where it spread to the surrounding countries (Boddupalli et al., 2020; Mahuku et al., 2015; Redinbaugh and Stewart, 2018). MLN symptoms include yellowing and drying of the leaves from the edges, stunting, and premature plant death (Figure 3) (Wangai et al., 2012).

MLN is caused by a combination of two plant viruses: maize chlorotic mottle virus (MCMV) from the *Tombusviridae* family and any of the viruses from the family *Potyviridae* (Niblett and Claflin, 1978). Some examples of the potyviruses are sugarcane mosaic virus (SCMV), maize dwarf mosaic virus (MDMV), or wheat streak mosaic virus (WSMV). As SCMV is endemic globally, MLN outbreaks in Africa could be attributed to the emergence of MCMV.

The potyviruses in dual-viral synergistic diseases have been reported to promote accumulation and symptoms of the heterologous virus through compromising the plant’s defense machinery (Pruss et al., 1997; Fedorkin et al., 2000; González-Jara et al., 2005; Ivanov et al., 2016). This characteristic has also been observed for MLN. Co-inoculation with SCMV caused a two-fold higher accumulation of MCMV in the plant cells as compared to MCMV inoculation alone (Xia et al., 2016).

Recent studies using next-generation sequencing suggest, however, that MCMV alone might be sufficient in causing MLN (Wamaitha et al., 2018; Mwatuni et al., 2020). In plants from geographically distant counties (Bomet, Kajiado, and Machakos) in Kenya, several plants that exhibited severe MLN symptoms tested positive only for MCMV as studied with next-generation sequencing and RT-PCR and showed no trace of SCMV. In another set of plants that exhibited MLN symptoms, another virus, maize streak virus, which belongs to the family *Geminiviridae*, was also detected along with MCMV, but no SCMV was detected. SCMV may lower the threshold of intrinsic defense in partially resistant plants to an extent where MCMV could readily replicate and spread. In genotypes with a weaker constitutive defense, MCMV alone might be sufficient to cause MLN.

The sudden appearance of MLN severely affected maize production in eastern Africa. The disease could destroy 20–100% of the crop. In 2013, it was estimated to have reduced maize production by half a million tons in Kenya (De Groote et al., 2016). The disease continues to threaten the maize crop in eastern Africa (Boddupalli et al., 2020). In addition to the resource-poor farmers, MLN has affected the small and medium companies involved in seed production and sales and processing of the grain. Naturally, there is a strong demand for MLN resistant hybrids.

#### MLN Resistance

The molecular mechanism of exactly how the causal viruses (MCMV and SCMV) together cause the MLN disease is not yet known. Regardless, if a large-effect QTL for MLN resistance is identified and validated, then it could be fine-mapped to identify the underlying gene. Genome editing could recreate appropriate favorable polymorphisms in the MLN-susceptible maize lines. However, the challenge is to edit the exact causal allele in the susceptible but elite maize lines, especially parents of popular commercial hybrids in Africa, and create resistant versions directly. Another potential route is to use a translational approach: identify maize orthologs of the genes for virus resistance from other crop species and edit them to determine whether they confer resistance against MLN.

Conventional plant breeding involves crossing an elite, commercial line (as a recurrent parent) to a donor parent (with MLN resistance) and then backcrossing over many cycles to recover the recurrent parent genome while introgressing the trait of interest from the donor parent. Backcrossing is a resource-intensive and time-consuming process. Even after eliminating a substantial proportion of the donor genome in the converted elite line, some of the not-so-desirable genes from the donor parent continue to be present, leading to unpredictable effects on agronomic performance. For example, after four backcrosses, ∼3% of the donor genes continue to persist in the converted line (Dhugga, 2022). Genome editing directly in elite, commercial but susceptible lines could eliminate the need for continued backcrossing, saving resources, speeding up product delivery, yet at the same time sparing the converted line the yield drag that accompanies backcrossing.

**FIGURE 4:**
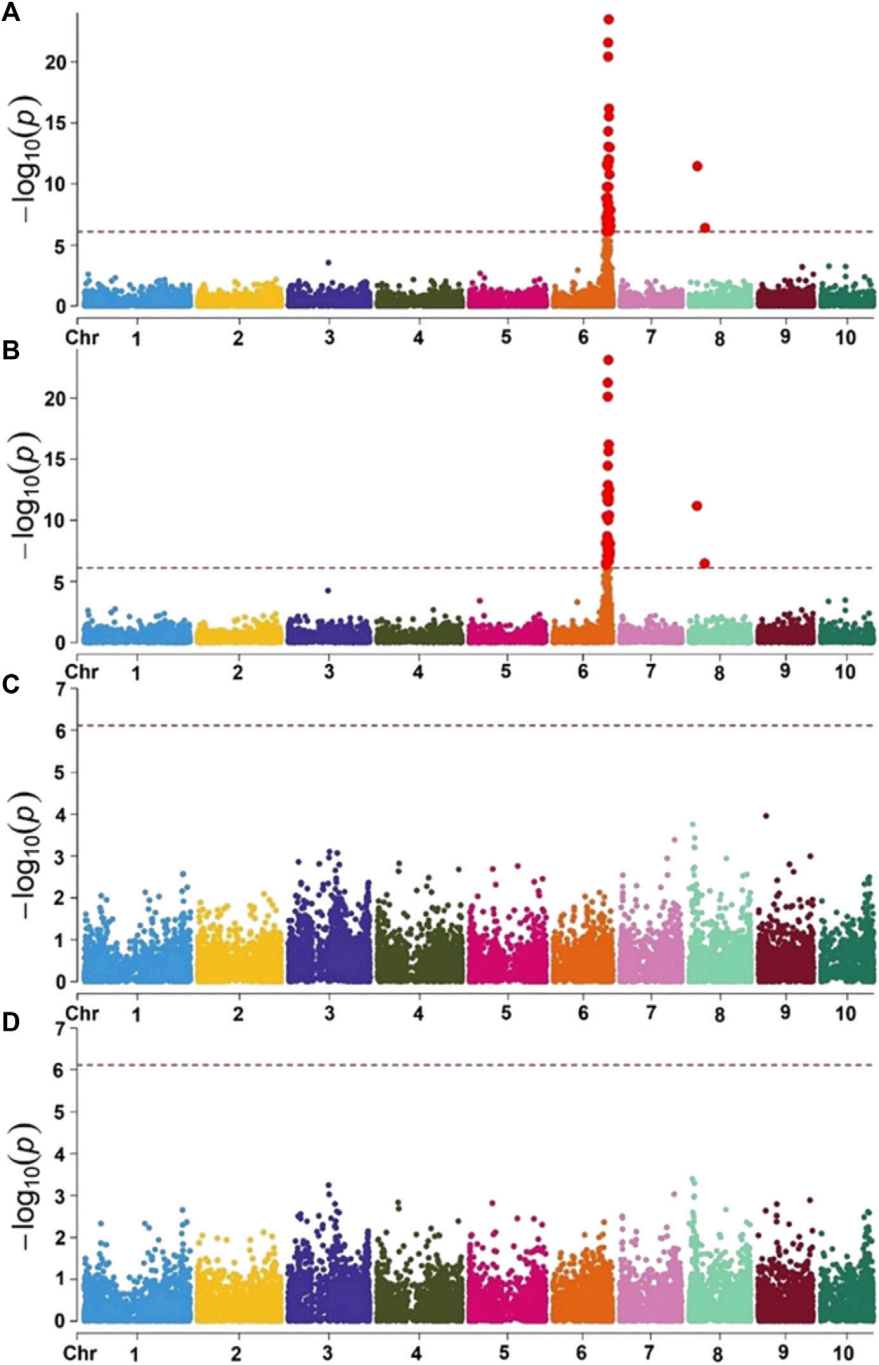
Mapping of QTL for resistance against maize lethal necrosis in CIMMYT germplasm. Figure reproduced from Murithi et al., 2021. Manhattan plot of GWAS using MLM in the selective genotyping populations. Combined genome-wide association scan for MLN disease severity (MLN_DS) **(A)** and the area under the disease progressive curve (AUDPC) values **(B)** based on the first three F2 populations (selective genotyping - SG) with KS23 background. Manhattan plots for MLN_DS **(C)** and AUDPC values **(D)** based on two F2 populations (CML494 X CZL068 and DTP-F46 X CML442) with no KS23 background. The horizontal dotted line indicates genome-wide significance and the plots above the line represent SNP markers that showed significance above threshold of *p* = 5 × 10^−7^.

As most of the hybrids grown in Africa are three-way crosses, all three lines for each hybrid would require the introduction of the recessive resistance allele. CIMMYT focused on a large-effect QTL for resistance against MLN from an exotic maize line, KS23-6, which was validated in several populations derived from its crosses with CIMMYT lines (Murithi et al., 2021). The QTL mapped near the telomere of the long arm of chromosome 6 (Figure 4). In partnership with Corteva Agriscience and under a grant from Bill and Melinda Gates Foundation (BMGF), CIMMYT has fine mapped this QTL to a ∼100 kb genetic interval (Boddupalli et al., 2020). Recessive inheritance suggests either a loss of function, which could result from an inactive form of the corresponding protein required by the virus for its replication or movement, or an altered protein sequence with a different conformation, which the virus is unable to recognize.

Maize lines susceptible to MLN were edited using CRISPR/Cas9 technology targeting the candidate genes for MLN resistance. After an outcross to the unedited parent and simultaneous screening for unintended changes in the genome with highly sensitive molecular tools, the lines are ready to be tested in the field (Zastrow-Hayes et al., 2015). The major QTL for MLN resistance identified in KS23-6 could act as a background source of resistance or could be directly created in other genetic backgrounds to further fortify their partial MLN resistance.

Deletion of various parts of the 100 kb genetic interval where the KS23-6 QTL for MLN resistance resides should allow identification of the causal polymorphism. Once validated, the causal gene could be directly edited in the CIMMYT lines, followed by reconstitution of the original three-way hybrids.

A translational approach by transferring knowledge on virus resistance from other plant species to maize is another avenue to improve resistance against MLN. Viruses are known to hijack their host’s protein translation machinery to replicate themselves. The host could become resistant if one or more of the components that virus hijacks is mutated (Sanfacon, 2015; Miras et al., 2017). Eukaryotic translation initiation factors (eIF) and two types of elongation factors have been reported to be recruited by the viruses to translate their RNA in plant species (Riis et al., 1990; Jackson et al., 2010). Mutants in several of them are known to confer resistance (Sanfacon, 2015). No *eIF* has yet been reported for virus resistance in maize, which has more than 25 *eIF* genes. An option is to use phylogenetic analysis to identify the maize orthologs of the genes from other plant species where they are known to be involved in virus resistance and then knock them out using SDN1 to determine whether and which ones confer resistance to MLN.

Tropical maize lines require 5–6 months in the greenhouse for each generation. In contrast, a fast-flowering line, referred to as mini-maize, cycles from seed to seed in 2 months (McCaw et al., 2016). Mini-maize is highly susceptible to MLN, providing a tool to expeditiously test genes for MLN resistance. Once the efficacious genes are identified, they could be altered directly in commercial CIMMYT lines.

### Sorghum

Sorghum (*Sorghum bicolor*) is the second most important cereal after maize in semi-arid and tropical areas of Africa. The demand for this crop is continuously increasing, as reflected by the increasing area under its cultivation trend. However, the current sorghum production cannot fulfill the increasing demand. The yields are low due to low-input farming systems, extreme environmental conditions, and limited crop improvement efforts in sorghum compared to other cereals.

#### Striga Resistance in Sorghum

Striga is a genus of parasitic plants that significantly limits agricultural production in Africa. Particularly, two species, *S. hermonthica,* and *S. asiatica*, limit sorghum and other cereal production in SSA. Currently, about two-thirds of the farmland under cultivation is infested with one or more species of Striga, directly impacting over 300 million peasant farmers in over 25 countries with yield losses of USD 7 billion annually (Ejeta, 2007).

The parasite is hard to manage because of its well-adopted parasitic lifestyle (Runo and Kuria, 2018). Current control strategies used for *Striga* management are based on cultural practices (e.g., crop rotation, intercropping/trap crops, different planting techniques, hand weeding, management of soil fertility), use of herbicide containing seed dressing, and direct chemical treatment of soil to reduce seed levels in the soil, and identification of resistant or tolerant varieties (Kanampiu et al., 2018). These methods are either ineffective or too expensive for smallholder farmers in Africa. Experts agree that effective control of parasitic plants can only be achieved through an integrated approach that highly exploits host-based resistance (Mwangangi et al., 2021). Modern techniques make such an approach feasible because of the increasing knowledge of the genetic mechanisms that underpin Striga-host interactions. Such advances can be combined with versatile genome editing tools to introduce host-based resistance against the parasite.

In our view, current opportunities for controlling Striga using genome editing point to three possibilities that hinge on interfering with the communication exchange that is intricately coupled between Striga and its hosts. The first approach could be developing Striga resistance by uncoupling Striga-sorghum interactions during germination and haustorium formation. Striga’s life cycle begins with germination induced by chemical cues produced by the host root exudate called strigolactones (Matusova et al., 2005; Gomez-Roldan et al., 2008). Subsequently, the parasite develops a specialized organ (haustorium) using chemical cues from the host referred to as haustorium inducing factors (HIFs) (Bandaranayake et al., 2010; Wada et al., 2019). Host triggered germination and haustorium induction represent the pre-attachment stages of the parasite, and hosts that “disallow” these processes to occur are said to harbor pre-attachment Striga resistance. The genetic cause of pre-attachment resistance in sorghum is due to mutations on the *LOW GERMINATION LOCI 1* (*LGS1*). Loss of function *lgs1* mutants are not effective in stimulating seeds of the parasite to germinate. Following on this information, gene-edited sorghum varieties were developed using CRISPR/Cas9 targeting the *LGS1.* The *lgs1* mutants showed high levels of resistance against the parasite (Bellis et al., 2020).

Aside from the *LGS1* loci in sorghum, other genes in the strigolactone biosynthetic pathway can be used to disrupt the flow of information from the host to the parasite. For example, (Bari et al., 2019) developed a tomato variety resistant to obligate root parasite *P. aegyptica*, by modifying the *carotenoid cleavage dioxygenase 8* (*CCD8*), critical in the strigolactone biosynthesis pathway. Obliteration of *CCD8* by CRISPR/Cas9 genome editing approach resulted in reduced production of orobanchol in tomato and subsequently less germination and resistance against the parasite. Similarly, (Butt et al., 2018) developed a rice variety using CRISPR/Cas9 with reduced *S. hermonthica* germination by targeting *OsCCD7*.

A second approach for Striga resistance could be uncoupling Striga-sorghum interactions mediated by host-derived susceptibility factors such as *DMR6*, a well-studied susceptibility gene in Arabidopsis (Damme et al., 2008). CRISPR/Cas9-mediated editing of the *DMR6* gene in tomato, banana, and sweet basil showed broad-spectrum resistance against fungal and bacterial pathogens (de Toledo Thomazella et al., 2021; Hasley et al., 2021; Tripathi et al., 2021b). Mutants of *DMR6* display reduced susceptibility to the pathogen. Based on these observations, one can hypothesize that Striga resistance can be achieved through uncoupling critical Striga-host interactions mediated by compatibility factors. Such a hypothesis is supported by recent findings that showed significant associations of sorghum resistance against Striga in a single nucleotide polymorphism on sorghum’s ortholog of *AtDRM6* (Kavuluko et al., 2021).

**FIGURE 5:**
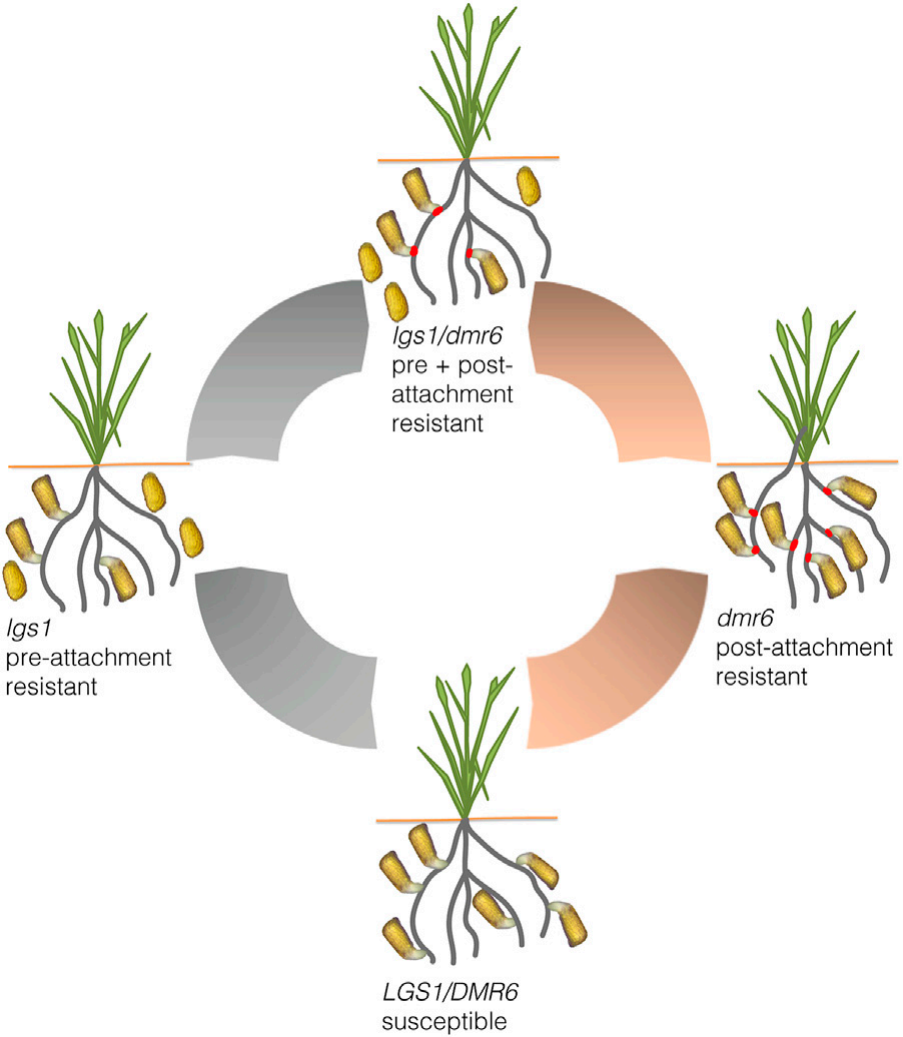
A schematic diagram summarizing potential genome editing approaches for Striga resistance in sorghum. Resistance can be imparted at the pre-attachment stage (gray arrows) by CRISPR/Cas9 mediated knock out of the *LOW GERMINATION LOCI I* (*LGS1*) to obtain *lgs1* edits that do not effectively stimulate parasite seed germination. And post-attachment resistance (peach arrows) can also be imparted by CRISPR/Cas9 mediated knock out of susceptibility genes such as *DOWNY MILDEW RESISTANT 6 (DMR6*) to create host-parasite incompatibility that inhibits unsuccessful parasite attachments (red spots). Both approaches can be used to develop multi-level resistance using breeding or CRISPR/Cas9 double knockouts of *LGS1* and *DMR6*.

Finally, it is desirable to stack multiple levels of Striga resistance for durable and broad-spectrum resistance (Figure 5). Preferably, such a strategy should target both stages of the Striga lifecycle: pre- and post-attachment resistance. To achieve stacked resistance, a two-pronged approach can be used: Firstly, by simultaneously targeting loci such as the *lgs1* and *dmr6* during genome editing, and secondly, by selecting and crossing products of two loci such as *lgs1* and *dmr6* editing. In any case, an initial detailed characterization of the mechanisms of resistance is critical.

#### Improved Nutrition Quality

Although sorghum is a staple food for millions of people in Africa, its grain has poor nutritional quality mainly because of low levels of essential amino acids, such as Lys, poor protein digestibility (Taylor and Schüssler, 1986; Aboubacar et al., 2001). Protein quality and digestibility is attributed to prolamins called kafirins, particularly the α – kafirin. Studies of the α-kafirin floury sorghum mutant P721Q, reveal that the genotype has low levels of α-kafirin and increased Lys (Oria et al., 2000; Wu et al., 2013). In addition, the reduced kafirin alters the protein structure to make it more accessible to gastric proteases, thereby increasing protein digestibility (Oria et al., 2000; Duodu et al., 2003; Wong et al., 2009). These studies have, therefore, made Kafirin a target for improving protein quality in sorghum. For example, RNA interference (RNAi) was used to reduce α-kafirins in sorghum (Kumar et al., 2012). More recently, a genome editing approach using CRISPR/Cas9 was used to create variants with reduced kafirin levels and improved protein quality and digestibility (Li et al., 2018).

### Wheat

Wheat is a source of carbohydrate and one of the main staple foods worldwide. The tetraploid durum wheat (*Triticum turgidum* ssp. *durum* L.) and the hexaploid bread wheat (*Triticum aestivum* L.) are the most widely grown types of wheat. Both species have highly conserved gene sequences and homoeologs among subgenomes, which allows CRISPR/Cas9 technology to simultaneously target mutations using single or multiple gRNAs (Smedley et al., 2021). The availability of reference genome sequences for different wheat cultivars has facilitated genome editing to modify several genes. However, the major challenge in wheat editing is that many elite cultivars are recalcitrant to *Agrobacterium-*mediated transformation. Nevertheless, several reports using TALENs and CRISPR/Cas9 have been published during recent past years, showing the usefulness of the genome editing system to generate the allelic mutation in wheat (Zhang et al., 2018; Matres et al., 2021). DNA-free genome editing technology has also been developed in wheat. The CRISPR/Cas9 RNA and CRISPR/Cas9 RNPs were delivered into wheat embryos by particle bombardment, and both methods created mutations in the target sites generating DNA-free genome-edited plants (Zhang et al., 2016; Liang et al., 2017). Recently, a combination of DNA-free genome editing, and base editing was reported in wheat with a frequency of C-to-T conversion of 1.8% (Zong et al., 2018). This technique would greatly facilitate the application of base and DNA-free editing in wheat improvement. The CRISPR/Cas9 technology has been applied to various gene targets of agronomically important traits.

#### Disease Resistance

The first demonstration of genome editing in wheat was achieved using TALENs to knockout three *TaMLO* homoeologs to create powdery mildew-resistant wheat (Wang et al., 2014). *MLO* is a susceptibility gene, and its loss of function confers durable and broad-spectrum resistance to powdery mildew in various plant species (Büschges et al., 1997). However, the knockout of the *MLO* gene can lead to growth penalties and yield losses. Recently, Li et al. (2022) demonstrated that *Tamlo*-R32, a mutant with a targeted deletion in the MLO-B1 locus, showed robust resistance to the powdery mildew disease in wheat without impacting its growth and yields.

In 2017, Zhang et al. (2017) reported using CRISPR to generate *Taedr1* wheat plants by simultaneously disrupting the three homoeologs of *EDR1*. The resulting mutant plants were resistant to powdery mildew and did not exhibit mildew-induced cell death (Zhang et al., 2017). Cytidine-deaminase-mediated base editing has been used in wheat to create herbicide-resistant plants by generating point mutations within the *acetyl-coenzyme A carboxylase* (ACC) gene (Li et al., 2018).

#### Improving Quality and Yields

CRISPR/Cas9 technology has been applied to wheat for improving grain quality. Low-gluten wheat was developed by targeting the *a-gliadin* genes (Sanchez-Leon et al., 2018). The wheat *gliadin* genes encode gluten proteins responsible for the celiac disease in genetically predisposed individuals. Later, wheat was edited for high-amylose targeting the *TaSBEIIa* gene (Li et al., 2020). The edited wheat line showed a significantly increased resistant starch content.

The yields of wheat have been improved by manipulating the negative regulatory genes. The genome-edited wheat developed by knocking out the three *TaGASR7* homoeologs (*TaGW2-A1*, *-B1,* and *-D1*), a gibberellin-regulated gene, showed a significant increase in kernel weight compared to the control wild-type (Zhang et al., 2016). Similarly, wheat mutants with a knockout of the *GW2* gene encoding a RING-type E3 ligase that controls rice grain weight demonstrated increased grain yields (Zhang et al., 2018).

### Yam

Yam (*Dioscorea* spp.) is a multi-species monocotyledonous crop widely cultivated in Africa, Asia, Oceania, and South America (Obidiegwu et al., 2020). In Africa, the primary yam growing areas consist of a six-country stretch referred to as the “yam belt” of West Africa. These countries include the Republic of Côte d’Ivoire, Ghana, Togo, Benin, Nigeria, and Cameroon and account for 92% of the global yam production (FAOSTAT, 2018). The yam crop plays a prime role in ensuring food security for West Africa, with its production in the region surpassing that of staples like maize, rice, and sorghum. Besides, yam is rich in bioactive phytonutrients that serve as excipients in the pharmaceutical industry, and the crop is integrated into the social, cultural, economic, and religious aspects of West Africans (Scarcelli et al., 2019; Ntui et al., 2021).

The production of this critical famine reserve crop is beset by numerous challenges, including susceptibility to pests and diseases, weed pressure, a poor yield capacity of local landraces, decreasing soil fertility, and scarcity of released accessions (Mignouna et al., 2008). At present, various yam accessions with superior qualities such as pest and disease resistance, improved organoleptic qualities, and broad environmental adaptability have been developed following substantial efforts in contemporary breeding. However, conventional breeding in yam has achieved extremely slow progress due to a lack of sufficient information on the yam genome and intrinsic attributes of the crop that lengthen the breeding cycle (Darkwa et al., 2020). Therefore, there is a need to supplement traditional breeding efforts with modern techniques that can bypass the current technical challenges and shorten the time taken to generate yam accessions with the desired phenotypic traits (Syombua et al., 2021). The recent release of the reference genome sequences of various *Dioscorea* species is expected to foster substantial advances in yam gene functional analysis to identify the genes controlling important traits and guide the precise modification of targeted traits in yam (Siadjeu et al., 2020).

The need for increased attention to crop improvement goes beyond the requirement of providing safe, nutritious, and sufficient food that meets the dietary requirements and food preferences of a growing global population (Prosekov and Ivanova, 2018).

Recently, a CRISPR/Cas9-based tool was developed for *Dioscorea rotundata* precisely targeting the *PDS* gene in the yam genome (Syombua et al., 2020). This study demonstrated that the mutation in the *DrPDS* gene generated complete albino plants with varying degrees of dwarfism. Sequence analysis revealed that the predominant mutation types in the two target loci were deletions and insertions.

**FIGURE 6:**
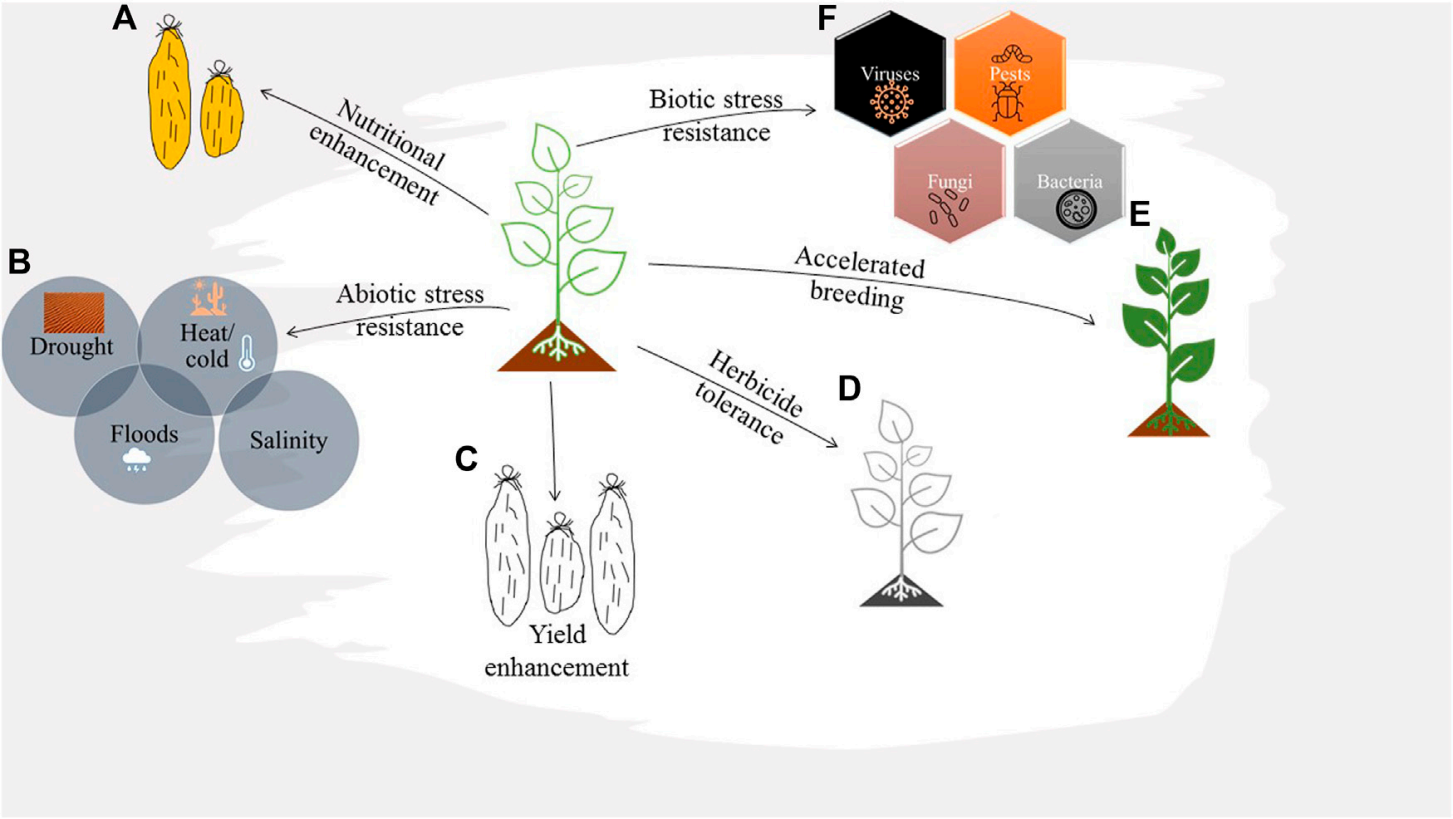
Schematic illustration of plant traits and genes that could be targeted by genome engineering for yam improvement. **(A)** Yam nutritional enhancement, e.g., increasing the beta carotene content by mutating the *Lycopene epsilon-cyclase* (*LCYE*) gene, or reducing post-harvest browning by targeting *polyphenol oxidase* genes (*PPO*s). **(B)** Engineering yams with improved resistance to abiotic stress, e.g., mutating ethylene response factors (*ERFs*) to improve the crop’s performance under stress conditions, upregulating the expression of anti-oxidative enzymes (*SOD*, *CAT*, *APX*, and *GPX*) or targeting genes that contribute to ROS redox balance such as Respiratory Burst Oxidase Homologue (*RBOH*) and *WRKY53* to increase ROS quenching capacity. **(C)** Improving the crop yield, e.g., mutating specific genes to modulate sink strength partitioning and promote sucrose translocation to the tubers, or enhancing photosynthetic rate by knocking out negative regulators of photosynthesis (*NRP*s) in the chloroplast and mitochondrion. **(D)** Engineering herbicide tolerance in yam plants for effective weed management and improved yields. **(E)** Accelerated yam breeding through allele replacement by homologous recombination-based knock in. **(F)** Enhancing biotic stress tolerance by mutating host susceptibility genes or upregulating the expression of disease resistance genes.

The study unlocked this orphan crop for more research towards its improvement for better productivity and reduced susceptibilities to biotic and abiotic stresses. Pests such as nematodes and diseases caused by fungi, bacteria, and viruses pose the most significant threat to global yam production, particularly in West Africa. Considering the associated economic losses, it is urgent to develop multidimensional strategies for combating this menace, as it also impedes the international exchange of the yam germplasm (Kenyon et al., 2008). Genome editing can be potentially applied in yam for mitigating the various challenges encountered in yam production and consumption (Figure 6) (Syombua et al., 2021).

#### Disease Resistance

Genome editing efforts towards developing disease-resistant crops have focused on gene disruption, replacement, or regulation. For instance, disrupting negative regulators of plant disease resistance such as host susceptibility (S) genes could offer durable resistance to diseases. This strategy could durably offer resistance to yam viruses by knocking out eukaryotic translation initiation factors (eIFs). In plants, eIFs are translation initiation factors that mediate the replication of plant RNA viruses, majorly potyviruses (Michel et al., 2019). Therefore, targeting these gene in yam could curb potyvirus infestations such as yam mosaic virus, as reported in cassava (Gomez et al., 2019) and cucumber (Chandrasekaran et al., 2016; Macovei et al., 2018). On the other hand, yam badnaviruses could be managed by targeting viral sequences integrated in host plant genomes, as demonstrated in banana (Tripathi et al., 2019b).

Susceptibility genes, mainly the mildew locus O (*MLO*), enhanced disease resistance *1* (*EDR1*), and the Non-Expressor of Pathogenesis-Related 3 (*NPR3*), could also represent good genome editing targets for generating resistance to fungal diseases in yam (Syombua et al., 2021). Since the fungal disease anthracnose is the most widespread foliar disease in yam (Amusa et al., 2003), developing endogenous resistance would alleviate the associated yield losses, ensuring food security for an economically disadvantaged population. Moreover genes that are highly conserved across plant species and have successfully generated plant fungal resistance, such as *WRKY* transcription factors and *ERF922,* could be precisely modified to achieve these endeavors (Noman et al., 2020). The precise modification of host susceptibility genes such as *DMR6* and *SWEET* could be affected to confer food yam with resistance to bacterial diseases. Further, the upregulation of host resistance genes is also a feasible approach for generating disease-resistant yam lines (Sameeullah et al., 2017).

#### Abiotic Stress Tolerance

Various abiotic stress factors, including declining soil fertility and unpredictable weather patterns such as floods, drought, and high temperatures, are critical constraints to sustainable yam production, resulting in substantial yield losses. Considering that yam growing regions span a wide range of agro-ecological zones that include marginalized lands (Frossard et al., 2017), there is a need to develop yam accessions with better adaptability to these conditions that can flourish in soils with reduced nutrient profiles. Gene function analyses in various crops have unveiled numerous differentially expressed genes following plant exposure to different abiotic stress conditions. In cassava, for instance, Ou et al. (2018) demonstrated that exposing cassava plants to high salinity levels, low-temperature pressure, and reduced water content upregulates the expression of *KUP* genes. Therefore, *KUP* and other gene orthologues in yam that are crucial for plant response to various stress conditions could be precisely modified to generate yam lines tolerant to stress conditions. Some of these genes include heat shock factors (*HSFs*), mitogen-activated protein kinases (*MAPK*s), ethylene response factors (*ERFs*), *MYB,* and *WRKYs* (Jaganathan et al., 2018; Syombua et al., 2021).

#### Weed Resistance

Yam production is generally a labor-intensive venture due to staking and weed competition requirements. Weeds directly compete with crops for access to light, water, and soil nutrients, thus reducing the yields. Besides, weeds could act as pathogen reservoirs, increasing the disease risk to yam plants. It is, therefore, important to develop environment friendly and economically sustainable strategies for weed management in yam plantations (Dentika et al., 2021). The development of gene-edited herbicide-tolerant yam accessions can effectively alleviate the weed challenge while alleviating crop phytotoxicity due to the reduced need for repeated use of chemical herbicides. Among the potential target gene orthologues in yam include the *EPSPS* (5-enolpyruvylshikimate-3-phosphate), *ACCase* (acetyl-coenzyme A carboxylase), *ALS* (acetolactate synthase) genes (Dong et al., 2021). As Syombua et al. (2021) discussed, genome engineering strategies also hold immense potential for improving the yield and nutritional quality of yams.

### Regulatory Landscape for Genome-Edited Products

The application of genome editing techniques is rapidly growing in agriculture. The global landscape of regulatory developments for genome-edited crops is quickly changing. Several countries have developed regulatory guidelines to handle genome-edited products, and several others are under discussion. However, there are differences among the countries regarding the regulation of genome-edited crop varieties. Genome-edited crop varieties with no foreign gene integration, mainly SDN1 type, are not regulated as GMOs in several countries (Tripathi et al., 2020; Entine et al., 2021). In 2015, Argentina endorsed the first regulation worldwide to establish a decision-making process for determining if genome-edited products should be regulated as GMOs or not on a case-by-case basis (Lema, 2019). According to their guidelines, genome-edited crops with no foreign gene are not subjected to GMO regulation. Later, several other countries in the region, such as Chile, Brazil, Colombia, Paraguay, Ecuador, Honduras, and Guatemala, adopted the same policies (Gatica-Arias, 2020).

Canada developed a product-based risk assessment framework based on the novelty of the products (Smyth, 2017). The novel crop varieties require additional regulatory oversight, regardless of whether they developed *via* mutagenesis, genetic engineering, or genome editing technologies. In the USA, no biosafety oversight of genome editing applications is required, if no genetic elements from pathogenic species or pesticidal traits are introduced. The three agencies [United States Department of Agriculture (USDA), Food and Drug Administration (FDA), and Environmental protection agency (EPA)] regulate the characteristics of the genome-edited products and not the process to develop them (Entine et al., 2021). Genome-edited crops lacking any foreign gene and that do not pose a risk to other plants and genome-edited food showing no food safety attributes different from those of conventionally bred crops are not subject to regulatory evaluation.

The Australian Office of the Gene Technology Regulator (OGTR) has technically amended the existing definitions of the GMO regulations to better address new breeding techniques applications (Office of the Gene Technology Regulator, 2018). According to the amendment, genome-edited crops with no foreign gene integration (SDN1) are not regulated in the same way as GMOs. The genome-edited products, where a repair donor template (i.e. SDN2 and SDN3 type) is used to guide editing, are treated as GMOs.

In Japan, the regulation of genome-edited products is based on the Japanese Cartagena Act (Tsuda et al., 2019). The Japanese government defined SDN-1 type genome-edited products as not representing “living modified organisms” based on the Japanese Cartagena Act. Japan considers crop varieties developed using genome editing with no new DNA as non-GMO. In 2022, China and India also published a new guideline for genome-edited crops. Several other countries, such as Philippines, are developing regulatory guidelines for genome-edited products.

Africa is also making progress in creating the enabling environment for the commercialization of genome-edited crop varieties. In Africa, Nigeria is the first country to publish the national biosafety guidelines for the regulation of genome editing (USDA, 2021). The Nigerian Biosafety Act defines GMO as “any organism living or non-living that possesses a novel combination of genetic material obtained using modern biotechnology” and Genome Editing as “a type of genetic engineering in which DNA is inserted, deleted, modified or replaced in the genome of a living organism.” Genome editing techniques may modify an organism’s genome in a way that results in a new combination of genetic material similar to GMO or results in organisms that are not genetically distinguishable from those developed from conventional breeding/natural selection. Therefore, Nigeria produces the regulatory guideline based on which genome editing and products thereof will be subject to appropriate Biosafety regulations on a case-by-case basis. Nigeria adopted an approach to regulate genome editing products, where the technique requires the use of recombinant DNA sequences or the genome-edited product has a novel combination of genetic material, the product will be regulated as GMO. However, the genome-edited products without any new combination of genetic material will be treated as non-GMO. The non-GMO edited products can be generated by not using recombinant DNA or using a recombinant DNA removed in the final product.

Kenya has recently developed genome editing guidelines as an important step towards the development of a genome editing regulatory framework in the country (ISAAA, 2022). Genome-edited products with deletions/knockouts without foreign DNA in the end-product, modifications made by inserting genes from sexually compatible species, and processed products whose inserted foreign genetic material cannot be detected, will not be regulated under the Biosafety Act. The decision on the genome-edited products will be made on a case-by-case basis. South Africa is under discussion for developing regulatory policies for genome editing. Other African countries, including Burkina Faso, Ghana, Ethiopia, Sudan, eSwatini, and Zimbabwe having GMO governance frameworks, started considering developing genome-editing policies.

Many countries are still in the process of developing regulatory guidelines for genome-edited products. There is a need for the coordination of regulatory approaches globally.

## Future Perspective

The field of genome editing has progressed through several phases, starting with oligo-mediated genome editing in the 1980s (Carroll, 2017). The main hurdle in the widespread adoption of genome editing was the low frequency of the edited events, which made progress painstakingly slow. CRISPR/Cas9 has revolutionized the field of genome editing because of its ease of use and high success rate (Carroll, 2017).

Traditional genome editing involves the delivery of the editing reagents into the plant cells through genetic transformation. In this approach, the editing reagents get integrated randomly into the plant genome and can therefore generate undesirable genetic changes. Moreover, integrating foreign DNA into plant genomes raises regulatory concerns as the edited plants may be considered GMOs. Accordingly, a DNA-free genome editing tool was developed to produce genetically edited crops without any foreign gene integration. This technique is accomplished using both protoplast-mediated transformation and particle bombardment. The delivery of CRISPR/Cas9 ribonucleoproteins (RNPs) into protoplasts was first demonstrated in Arabidopsis, tobacco, lettuce, and rice (Woo et al., 2015). Similarly, Malnoy et al. (2016) produced DNA-free grape and apple by delivering purified CRISPR/Cas9 RNPs into their protoplasts.

Genome editing has a prominent role to play in improving agriculture in Africa. Many researchers are exploring the potential of genome editing in developing crop varieties for a better and more sustainable African Agriculture. However, it requires adequate funding and enabling policies to release genome editing products.

Since the CRISPR/Cas9-mediated editing develops an improved crop variety by modifying its endogenous genome through deletions, insertions, or substitution, or even inserting or replacing a full-length gene from the same plant species at the targeted site in a very precise manner, these edited varieties are free from foreign gene and need not go through a complex and time-consuming biosafety regulation similar to GMOs for commercialization. The genome-edited crop varieties lacking any foreign gene remain indistinguishable from those developed through conventional breeding. Genome-editing products, particularly SDN1 type, with gene knockouts but with no foreign gene integration, are not regulated as GMO in several countries, including two countries in Africa, Kenya and Nigeria.

In summary, CRISPR-mediated editing has the potential to improve crops with disease resistance, abiotic stress tolerance and improved nutritional content. Several products, such as disease-resistant banana, MLN-resistant maize, and Striga-resistant sorghum, are in the pipeline and closer to being ready for release in Africa. The CRISPR-based genome editing tool is considered as one of the powerful technologies for improving agriculture to feed the rapidly growing population. It can develop genome-edited crop varieties with no foreign-gene integration like those created through conventional breeding.
